# Is There a Reversal in the Effect of Obesity on Mortality in Old Age?

**DOI:** 10.4061/2011/765071

**Published:** 2011-09-28

**Authors:** Jiska Cohen-Mansfield, Rotem Perach

**Affiliations:** ^1^Department of Health Promotion, School of Public Health, Sackler Faculty of Medicine, Tel Aviv University, P.O.B. 39040, Ramat Aviv, Tel Aviv 69978, Israel; ^2^Herczeg Institute on Aging, Tel Aviv University, P.O.B. 39040, Ramat Aviv, Tel Aviv 69978, Israel; ^3^George Washington University Medical Center and School of Public Health, Washington, DC 20037, USA

## Abstract

Studies of obesity and its relationship with mortality risk in older persons have yielded conflicting results. We aimed to examine the age-related associations between obesity and mortality in older persons. Data were drawn from the Cross-Sectional and Longitudinal Aging Study (CALAS), a national survey of a random sample of older Jewish persons in Israel conducted during 1989–1992. Analyses included 1369 self-respondent participants aged 75–94 from the Cross-Sectional and Longitudinal Aging Study (CALAS). Mortality data at 20-year followup were recorded from the Israeli National Population Registry. Obesity was significantly predictive of higher mortality for persons aged 75–84, but from age 85 onwards, obesity had a protective effect on mortality albeit at a nonsignificant level. Being underweight was consistently predictive of mortality. Findings suggest that the common emphasis on avoiding obesity may not apply to those advancing towards old-old age, at least as far as mortality is concerned.

## 1. Introduction

Increased obesity rates comprise a major public health concern over the world [1, 2]. While obesity was associated with increased mortality risk for young and middle-aged adults [3, 4], studies of older persons have yielded conflicting results. Some indicate higher mortality rates for obese older persons [4–6], while others found no such associations [7–9] or evidence for a reversed relationship, that is, linking decreased mortality with higher Body Mass Index (BMI) values [9–13].

Studies of the relationship between obesity and mortality among older persons suggest the impact of obesity varies according to age. In a study of persons aged 44–101 with 23 years of followup, obesity increased mortality only among persons under 75 years of age [5]. Similarly, a study of adults aged 30 and over with a 12-year followup found higher mortality rates for obese younger persons, but not for those aged 75 and over [14]. In line with that, higher BMI was associated with lower mortality among persons aged 70–88 [9], among older persons aged 70 and older [12], and among persons aged 75–89 [10]. 

This paper addresses the following question: what are the age-related associations between obesity and mortality in older persons? Accordingly, we examine the impact of obesity on mortality in persons who survived to old-old age, that is, persons over 75 years of age.

## 2. Methods and Procedures

### 2.1. Participants and Procedure

The sample was part of the Cross-Sectional and Longitudinal Aging Study (CALAS). The CALAS conducted a multidimensional assessment of a random sample of the older Jewish population in Israel, stratified by age group (75–79, 80–84, 85–89, and 90–94), gender, and place of birth (Asia-Africa, Europe-America, Israel). Data collection took place during 1989–1992. The inclusion criteria were being self-respondent, community dwelling or a nursing home resident, and between 75–94 years of age. More information regarding the CALAS appears in various publications [15–17]. The CALAS was approved for ethical treatment of human participants by the Institutional Review Board of the Chaim Sheba Medical Center in Israel. The present analyses include 1369 self-respondent participants aged 75–94. The mean age was 83.52 (SD = 5.42).

## 3. Measures

*Sociodemographics *include age, gender, place of birth (Israel, Middle East/North Africa, Europe/America), having children (number of children alive and number of children deceased), education (number of years of education), and financial situation (having additional income beyond social security, 0 = no, 1 = yes). Smoking status was measured by two items, based on the EPESE questionnaire [18]: Do you smoke (yes, no); Did you smoke in the past (yes, no).

### 3.1. BMI

The interviewer measured the participant's weight and height, and Body Mass Index was calculated (<22 = underweight, 22–30, >30 = obese) [19].

### 3.2. Mortality Followup

Mortality data within 20 years from the date of sampling were recorded from the Israeli National Population Registry (NPR). Of the original sample, 59 participants were still alive. Hence we have complete mortality data on 95.1% of the sample.

## 4. Statistical Analysis

The impact of obesity on 20-year mortality was tested using Cox regression models. In order to examine whether obesity had an effect on mortality, we examined the impact of obesity on mortality after controlling for stratification variables (age, gender, origin), as well as for education, financial status, and having children. The impact of obesity as well as underweight was examined. The same model was examined separately for males and females and separately for ages 75–84 and for ages 85–94. Each analysis was conducted twice, once with community-dwelling participants and once with the full sample, including nursing home residents.

## 5. Results

Descriptive information on background variables and BMI for both the full sample and community dwellers is presented in Table 1.

**Figure 1:**
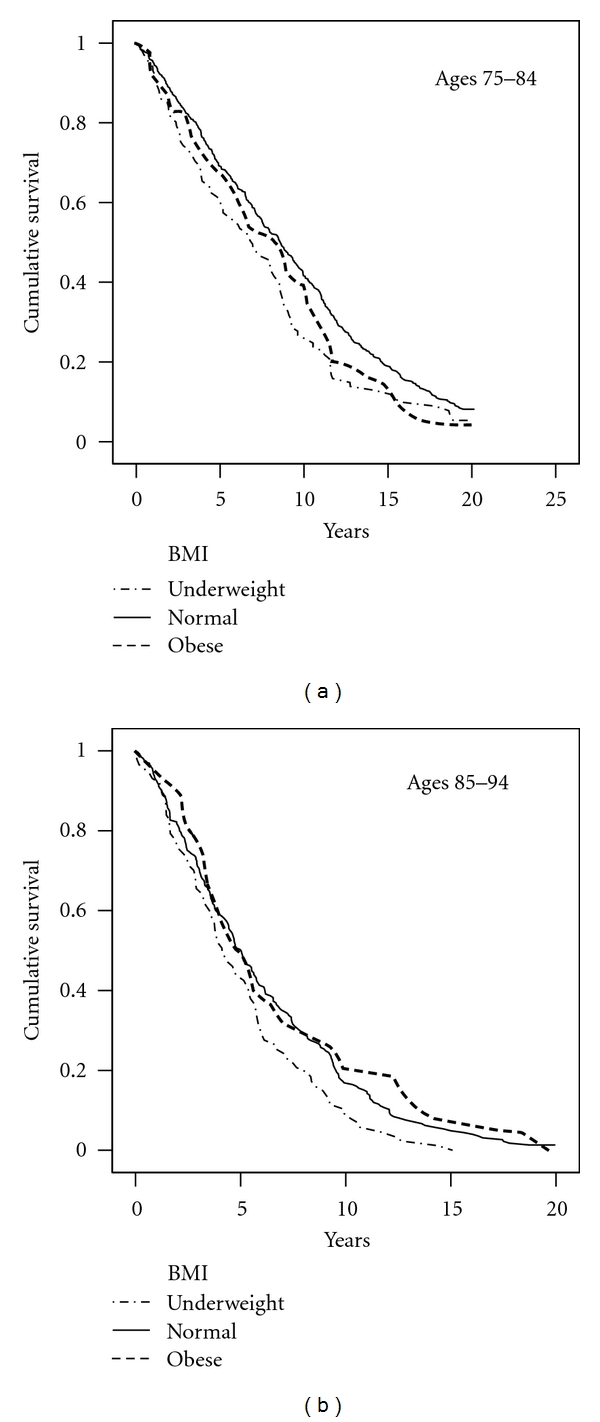
Kaplan-Meier Survival Curves according to Body Mass Index (BMI) in community dwellers. Obesity was significantly predictive of higher mortality for persons aged 75–84, but not for 85–94. Being underweight was consistently predictive of mortality. BMI < 22 = underweight, 22–30 = normal, >30 = obese.

While underweight was significantly predictive of mortality under all conditions examined (total population, for each gender, and each age group), obesity was not significantly predictive of mortality when the whole population was examined (Table 2). However, when participants were divided by age, obese persons aged 75–84 had a significantly higher mortality Hazard Ratio (HR) than those with normal weight for the total sample (HR = 1.296, Confidence Interval [CI] = 1.026–1.659, *P* = .030) and for community dwellers (HR = 1.320, CI = 1.036–1.683, *P* = .025). In contrast, obese persons aged 85–94 had a lower, albeit nonsignificant risk ratio for the total sample (HR = .944, CI = .703–1.268, *P* > .05) and for community dwellers (HR = .854, CI = .616–1.185, *P* > .05), indicating that at this age obesity no longer posed a greater risk of mortality. When dividing participants by gender, no significant impact for obesity on mortality was found. Upon adding smoking status (ever versus never) as a covariate to the analysis, the results were nearly identical (see Table 3); smoking status was not significant when included with all the other covariates, but BMI was. The findings concerning the relationship between BMI and 20-year mortality among community dwellers aged 75–84 and 85–94 are illustrated in the survival curves in Figure 1.

## 6. Discussion

We investigated the relationship between obesity and mortality in a random sample of older Israelis, with all participants having survived to at least 75 years old at baseline. Obesity was predictive of mortality for those aged 75–84 years, but from age 85 onwards, obesity had a protective effect on mortality albeit at a nonsignificant level. When examining the entire sample (ages 75–94), obesity was not predictive of mortality. Being underweight was consistently predictive of mortality. 

 Our findings linking obesity to increased mortality rates among older persons aged 75–84 support those of Ajani et al. [4], reporting greater mortality risk as BMI increased among men aged 70–84 and those of De Gonzalez et al. [20], reporting increased all-cause mortality rates in white never-smokers aged 70–84 with BMI over 30 in a median 10-year followup. Because a variety of age groups within old age have been previously studied, comparing findings is somewhat problematic. Yet, current findings are in discord with some previous evidence. For example, no excess mortality was associated with obesity for persons aged 75–84 [14] in a convenience sample of 62,116 men and 262,019 women from the American Cancer Society's Cancer Prevention Study I. Generalizability of these results is limited due to numerous exclusions (e.g., including only never smokers, only white ethnicity). In a slightly older age group, a protective effect of obesity was found, linking it to lower mortality in a sample of 470 in-patients hospitalized for acute illness aged 75–89 [10]. Similarly, decreased mortality rates were associated with higher BMI among persons of 70–88 years of age [9]. Considering the age range in these studies, it may be that a shift in the relationship between obesity and mortality takes place between age 80 and 90.

Supporting the latter notion, obese persons aged 85–94 in the current study had a lower, though not statistically significant, mortality hazard ratio, in contrast with the significantly higher mortality risk of those 75–84 years old. Previous investigations of mortality and obesity among persons over 80 [5] and over 85 years of age [14] yielded no significant associations between the two. 

The relationship between obesity and mortality in older persons is controversial (see [21]). In a review of that relationship [22], obesity was associated with elevated mortality risk in most studies. In line with that, studies on the effect of weight loss in older persons support its favorable health outcomes, even regarding minor amounts of weight loss [21]. These benefits include reducing blood pressure and the severity of diseases such as osteoarthritis and non-insulin-dependent diabetes mellitus [23]. In contrast, inverse associations between BMI and long-term mortality in persons with Chronic Heart Failure (CHF) have been reported in a number of studies [24–29], in what was termed “the obesity paradox” [28]. Following that notion, increased BMI was linked to decreased mortality in hospitalized persons aged 75–89 [10]. Current findings suggest a possible direction for disentangling the obesity paradox, in that the impact of obesity on mortality in old age was age-dependent, shifting from detrimental to favorable between age 80 and 90. In support of that, the concepts of frailty and disability in old age have been proposed to be age-sensitive [30]. It may be that as one gets older, the protective effects of obesity become more pronounced. Lower rates of osteoporosis in heavier persons, possibly due to greater weight-bearing bone formation [31], may reduce their risk of falls and subsequent potential trauma. Obesity may also provide energy reserves in times of stress, illness, and trauma [32, 33]. In addition, obesity may prolong the period of predeath weight loss, as aging is associated with decreased food intake [34]. A progressive drop in BMI was found in a longitudinal study following a small sample of healthy Okinawan centenarians (aged at least 99 years at baseline) until death between ages 110–112, thus suggesting that BMI decline at very old ages may signal sarcopenia, frailty, and/or underlying subclinical pathology [35]. Decreased mortality among persons with obesity in very old ages may reflect a selective survival effect [36, 37] whereby persons who are more prone to the adverse health outcomes of obesity due to the effect of genetic or environmental factors suffer from higher mortality in middle age, which leaves a more resilient overweight older population [11]. Another possible explanation is that of a ceiling effect, as absolute mortality long-term risk increases with age and eventually converges, regardless of any health-associated risk levels [11, 30]. 

Evidence regarding the most effective means for achieving weight loss in older persons is controversial, with modest positive outcomes reported for interventions involving diet, physical exercise, and a combined approach (see [38, 39]). For example, a high saturated fat and no-starch diet yielded weight loss without adverse effects on lipids in persons aged 53–73 after 6 weeks [40]. In another study, regular physical exercise for a period of 12 months successfully reduced body weight and body fat in overweight and obese women aged 50–75 [41]. However, in a study of obese and overweight persons with knee osteoarthritis aged 60 and over, the combination of diet and exercise interventions over a period of 18 months provided better overall improvements in measures of pain, function, and mobility, compared with each modality alone [42]. Nonetheless, current knowledge on interventions aimed at weight reductions in the older population is limited, particularly with regard to mortality outcomes [38]. Future studies of that population which assess interventions' effect on mortality are needed. 

One limitation of the study involves the use of BMI as a single indicator of body fat. In adults with BMI within the range of 25 to 34.9, BMI fails to provide an adequate biomarker of body fat [43]. In addition, increased mortality due to overweight in older persons is largely affected by a high rate of chronic disease and diminished lean mass [44]. Previous investigations support the value of examining additional body habitus indices. Specifically, lean mass and Lean Mass Index (LMI) predicted all-cause mortality in older Asian persons over 65 years of age, while BMI did not [45]. Similarly, Waist Circumference (WC), as opposed to BMI, predicted mortality at 12-year followup in older persons with CHF and more modestly so among those without CHF [46]. Indeed, public health guidelines indicate the consideration of WC when assessing health risks in overweight adults [43]. Finally, the reported adverse effects associated with visceral fat on health conditions including cardiovascular disease, insulin resistance, and metabolic syndrome [47, 48] support the use of anthropometric measures of abdominal obesity in assessing obesity in older persons [46]. Due to our use of an existing dataset, we were unable to examine the associations between mortality and other measures such as chronic disease, lean mass parameters, and WC. Future studies may benefit from the inclusion of anthropometric measures.

In sum, with the increasing numbers of old-old persons and of their life expectancy, extra attention is often given to avoiding obesity. Current findings suggest that such an emphasis may not apply to those advancing towards old-old age, at least as far as mortality is concerned. As, unlike in some previous investigations of obesity in old age, the current study involved a national representative sample, the results may be more generalizable. While our data included participants as old as 94 at baseline, future research could examine the impact of obesity on mortality when following even older ages.

## Figures and Tables

**Table 1:** Sample characteristics.

	Total (N = 1369)	Community dwellers (*n* = 1200)
	M (SD)/%	M (SD)/%
Age	83.52 (5.42)	83.10 (5.32)
Gender (female)	46.3	44.9
Place of birth		
Europe/America	37.5	37
Middle East/N. Africa	30.6	32.7
Israel	31.8	30.3
Education (*n* = 1310/1149)	7.79 (5.54)	7.63 (5.51)
Financial status		
(no additional income)	39.4	40.3
(*n* = 1312/1155)		
Having children (yes)	90.5	91.6
BMI		
Obese (>30)	11.8	11.9
Underweight (<22)	15.8	15.7
(missing)	16	14.7

**Table 2:** Cox Regression Models Predicting Mortality at 20-year followup by BMI, and age.

	Full sample				Age groups							
					75–84 year olds				85–94 years old			
	CD and NH residents		CD		CD and NH residents		CD		CD and NH residents		CD	
	*N* = 1369		*n* = 1200		*n* = 809		*n* = 752		*n* = 560		*n* = 448	
	HR	CI	HR	CI	HR	CI	HR	CI	HR	CI	HR	CI

Background												
Age	1.068^c^	1.055–1.081	1.066^c^	1.052–1.080	1.074^c^	1.044–1.105	1.073^c^	1.043–1.105	1.062^b^	1.025–1.100	1.065^b^	1.025–1.107
Gender (male=1)	1.31^c^	1.151–1.492	1.307^c^	1.138–1.502	1.50^c^	1.266–1.777	1.518^c^	1.272–1.813	1.093	.887–1.346	1.049	.834–1.318

Origin												
East	.857	.722–1.017	.874	.729–1.048	.972	.777–1.216	1.006	.799–1.268	.731^a^	.559–.955	.715^a^	.533–.958
West	1.032	.889–1.199	1.043	.888–1.225	1.027	.849–1.244	1.038	.848–1.270	1.028	.805–1.313	1.034	.786–1.359
Years of education	.995	.983–1.008	.996	.983–1.010	.997	.980–1.015	.999	.981–1.017	.992	.974–1.011	.993	.972–1.015
Additional income	.940	.824–1.071	.933	.812–1.072	.845^d^	.711–1.003	.858	.718–1.026	1.040	.845–1.279	.993	.791–1.247
Had children	.702^b^	.568–.868	.680^b^	.535–.863	.657^b^	.501–.861	.622^b^	.464–.835	.791	.554–1.129	.780	.506–1.204

BMI												
Obese (>30)	1.117	.930–1.342	1.091	.898–1.326	1.297^a^	1.026–1.639	1.320^a^	1.036–1.683	.944	.703–1.268	.854	.616–1.185
Underweight (<22)	1.414^c^	1.202–1.665	1.409^c^	1.183–1.677	1.278^a^	1.014–1.611	1.317^a^	1.036–1.684	1.474^b^	1.162–1.870	1.400^a^	1.075–1.824
*χ* ^2^ (df = 2)	16.511^c^		13.95^b^		7.374^a^		8.178^a^		10.855^b^		8.188^a^	

^a^ *P* < .05, ^b^ *P* < .01, ^c^ *P* < .001.

^d^.1 > *P* > .05.

CD= Community Dwellers; NH= Nursing Home; HR= Hazard Ratio= Exp (b); CI= Confidence Interval.

**Table 3:** Cox Regression Models Predicting Mortality at 20-year followup by BMI, and age, controlling for smoking status (Ever versus Never).

	Full sample				Age groups							
					75–84 year olds				85–94 years old			
	CD and NH residents		CD		CD and NH residents		CD		CD and NH residents		CD	
	*N* = 1369		*n* = 1200		*n* = 809		*n* = 752		*n* = 560		*n* = 448	
	HR	CI	HR	CI	HR	CI	HR	CI	HR	CI	HR	CI

Background												
Age	1.068^c^	1.055–1.082	1.067^c^	1.053–1.081	1.077^c^	1.047–1.108	1.076^c^	1.045–1.108	1.062^b^	1.025–1.100	1.065^b^	1.025–1.107
Gender (male = 1)	1.267^b^	1.104–1.453	1.265^b^	1.092–1.465	1.429^c^	1.195–1.709	1.449^*c*^	1.202–1.746	1.087	.871–1.357	1.051	0.823–1.342

Origin												
East	.849	0.715–1.008	0.867	0.723–1.040	0.960	0.767–1.201	0.993	.788–1.252	0.730^a^	0.559–0.955	0.715^a^	0.533–0.958
West	1.030	0.887–1.197	1.042	0.887–1.224	1.024	0.846–1.240	1.033	.844–1.264	1.028	0.805–1.313	1.033	0.786–1.358
Years of education	0.994	0.982–1.007	0.996	0.982–1.009	0.998	0.981–1.015	0.999	.981–1.017	0.992	0.974–1.011	0.993	0.972–1.015
Additional income	0.940	0.829–1.070	0.932	0.811–1.071	0.844^d^	0.711–1.003	0.858^d^	0.717–1.026	1.040	0.845–1.279	0.993	0.791–1.247
Had children	0.705^b^	0.570–0.872	0.684^b^	0.538–0.869	0.661^b^	.504–.867	0.629^b^	0.468–0.844	0.791	0.554–1.130	0.780	0.506–1.204
Smoking status (1= ever)	1.106	0.969–1.262	1.103	0.959–1.269	1.153^d^	0.974–1.364	1.151	0.966–1.371	1.015	0.816–1.263	0.993	0.781–1.263

BMI												
Obese (>30)	1.122	0.934–1.347	1.096	.902–1.332	1.291^a^	1.022–1.631	1.315^a^	1.032–1.676	0.946	0.704–1.273	0.853	0.614–1.187
Underweight (<22)	1.411^c^	1.199–1.661	1.404^c^	1.179–1.671	1.267^a^	1.005–1.597	1.304^a^	1.025-1.657	1.475^b^	1.163–1.871	1.400^a^	1.075–1.824
												
*χ* ^2^ (df = 2)	16.346^c^		13.699^b^		7.003^a^		7.789^a^		10.852^b^		8.185^a^	

^a^ *P* < .05, ^b^ *P* < .01, ^c^ *P* < .001.^d^.1 > *P* > .05.

HR= Hazard Ratio= Exp (b); CI= Confidence Interval.
